# Peripheral blood mononuclear cell transcriptomes reveal an over-representation of down-regulated genes associated with immunity in HIV-exposed uninfected infants

**DOI:** 10.1038/s41598-019-54083-4

**Published:** 2019-12-02

**Authors:** Zaneta D. Musimbi, Martin K. Rono, James R. Otieno, Nelson Kibinge, Lynette Isabella Ochola-Oyier, Etienne Pierre de Villiers, Eunice W. Nduati

**Affiliations:** 1 Center of Biotechnology and Bioinformatics, Chiromo Campus, University of Nairobi, Nairobi, Kenya; 2 KEMRI-Wellcome Trust Research Programme, Kilifi, Kenya; 3 Pwani University Biotechnology Research Centre, Pwani University, Kilifi, Kenya; 4 Centre for Tropical Medicine and Global Health, University of Oxford, Oxford, UK

**Keywords:** Molecular biology, Infectious diseases

## Abstract

HIV-exposed uninfected (HEU) infants are disproportionately at a higher risk of morbidity and mortality, as compared to HIV-unexposed uninfected (HUU) infants. Here, we used transcriptional profiling of peripheral blood mononuclear cells to determine immunological signatures of *in utero* HIV exposure. We identified 262 differentially expressed genes (DEGs) in HEU compared to HUU infants. Weighted gene co-expression network analysis (WGCNA) identified six modules that had significant associations with clinical traits. Functional enrichment analysis on both DEGs and the six significantly associated modules revealed an enrichment of G-protein coupled receptors and the immune system, specifically affecting neutrophil function and antibacterial responses. Additionally, malaria pathogenicity genes (thrombospondin 1-(*THBS 1*), interleukin 6 (*IL6*), and arginine decarboxylase 2 (*ADC2*)) were down-regulated. Of interest, the down-regulated immunity genes were positively correlated to the expression of epigenetic factors of the histone family and high-mobility group protein B2 (*HMGB2*), suggesting their role in the dysregulation of the HEU transcriptional landscape. Overall, we show that genes primarily associated with neutrophil mediated immunity were repressed in the HEU infants. Our results suggest that this could be a contributing factor to the increased susceptibility to bacterial infections associated with higher morbidity and mortality commonly reported in HEU infants.

## Introduction

Highly active antiretroviral therapy (HAART), is currently the most promising intervention for HIV control, particularly in the prevention of mother to child transmission (PMTCT). PMTCT has increased the number of HIV infected women who can safely have HIV uninfected infants. Despite escaping viral infection, the HIV exposed but uninfected (HEU) infants, have been shown to have an increased risk in mortality and morbidity^1,2^. The higher mortality is often due to the HEU infants’ increased susceptibility to infectious diseases, many of which prove difficult to treat^3^. Although the rates of diarrheal infections in HEU infants are similar to those in infants born to HIV negative mothers, commonly referred to as HIV-unexposed uninfected (HUU) infants, they are often more severe requiring hospitalization^4^. Furthermore, life-threatening bacterial infections with encapsulated pathogens, including *Haemophilus influenza* and *Streptococcus pneumonia*, are more common during the first twelve months of life in HEU infants^5–7^. The increased susceptibility of HEU infants to infections can partly be attributed to the lower levels of transferred protective maternal antibodies, inadequate parental care or exposure to maternal ailments such as tuberculosis^8^. Moreover, *in utero* exposure to HAART and HIV antigens may also be associated with adverse health outcomes such as mitochondrial dysfunction^9,10^, cardiac function and growth^11,12^ and an altered cytokine milieu leading to poor immune cell development and immune responses after birth^13,14^.

In comparison to HUU infants, HEU infants have previously been shown to have an enhanced expression of CD40L on activated T-lymphocytes^15^. In addition, HEU’s have higher numbers of CD3+ cells^16^, an intricate pattern of defects in CD4+ and CD8+ T-lymphocyte subpopulations, (which show a shift from naïve to memory phenotypes and an increase in peripheral immature T-lymphocytes^17,18^), altered dendritic cells^19^, a reduction in the proportion of circulating follicular helper T-cells^20^ and impaired progenitor T-cell function that leads to reduced thymic output and results in lower naïve CD4 counts^15,21^. Some of these T-cell parameters that are altered at birth are known to persist beyond the first year of life^17,18^. The B-cell compartment is also affected in HEU infants, albeit more subtly. Some studies have reported an increase in cord blood B-lymphocytes marked by higher numbers of CD19+/CD5+ cells^16^, a reduction in the resting memory B-cells (primarily due to changes in the unswitched memory B-cell subset^22^) and poorer humoral responses to a wide range of vaccines^15,17^.

These phenotypic, functional and clinical observations highlight a compromised immune system in HEU infants. Comparisons at the transcriptomic level can provide a robust and sensitive approach to identify subtle changes underlying biological and immune mechanism differences between HEU and HUU infants. In this study, we performed transcriptional analyses of peripheral blood mononuclear cells (PBMCs) from HEU and HUU infants using an RNAseq approach. We uncovered several HEU transcriptome markers and showed that the down-regulated genes in HEU infants are functionally related to diverse biological pathways with an over-representation of pathways associated with immunity.

## Results

### Baseline characteristics of the study population

Samples used in this study were previously collected from an established cohort of infants born to HIV-positive mothers^23^. A total of 19 HEU and 15 HUU infants were analysed. The median age of the HEU infants sampled at the early time point was 12.13 months (IQR [12.07–12.60]) and for the late time point, 18.9 months (IQR [17.95–21.13]). On the other hand, the median age of the HUU infants was 12.58 months (IQR [12.21–13.03]) and 16.56 (IQR [15.18–22.18]) for the early and late time points, respectively. There was no statistical difference (Mann Whitney nonparametric test) between the median age of the HUU and HEU infants at both the early (p = 0.60) and late (p = 0.20) time points, respectively. A comparison of the haematological parameters, in HEU and HUU infants, taken at the time of sample collection, showed no statistical differences in white and red blood cell counts, lymphocyte, platelets, neutrophil, monocyte and eosinophil counts (Supplementary Table S1).

### Differential expression of PBMC genes between HEU and HUU infants

**Figure 1:**
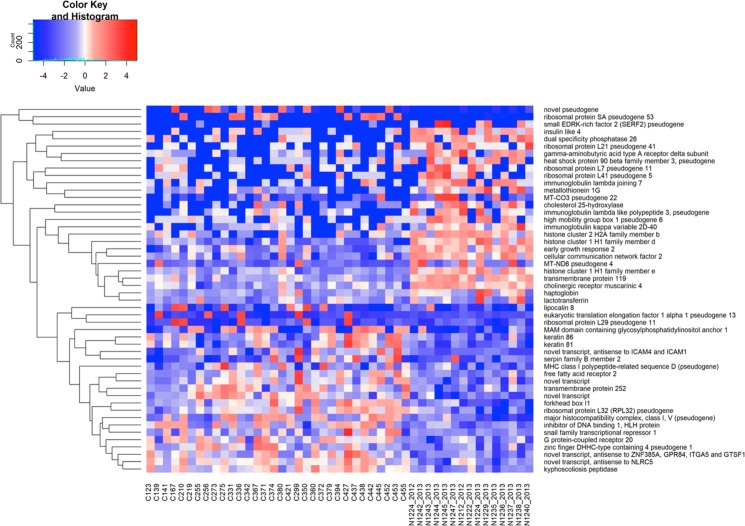
Hierarchical clustering of the top 50 differentially expressed genes (up- and down-regulated). Red; relative increase in gene expression, blue; relative decrease in gene expression. x-axis; sample identification, y-axis; genes identified.

To investigate gene transcription profiles, we sequenced mRNA extracted from PBMCs sampled at twelve (n = 18) and twenty-four months (n = 14) after birth from HEU infants and in HUU infants (n = 15). After quality control filtering, 47 transcriptomes with an average read depth of 30 million per sample were obtained (Supplementary Fig. S1). Differential gene expression analysis revealed a total of 262 differentially expressed genes (DEGs) of which approximately two thirds (188) were up-regulated, while a third (74) were down-regulated (Supplementary Fig. S2 & Table S2). The top 25 upregulated and downregulated DEGs are shown in Fig. 1a. Due to the age range around the targeted 12- and 24-months age groups, we analysed within these two populations genes showing significant transcriptional differences probably attributed to the infant’s age, henceforth referred to as HEU-DEGs. Comparisons between DEGs and HEU-DEGs showed an overlap of only 5% (Supplementary Fig. S3). Therefore, we concluded that DEGs were not related to the HEU infant’s age, but rather were due to differences between populations (HEU vs. HUU).

**Figure 2:**
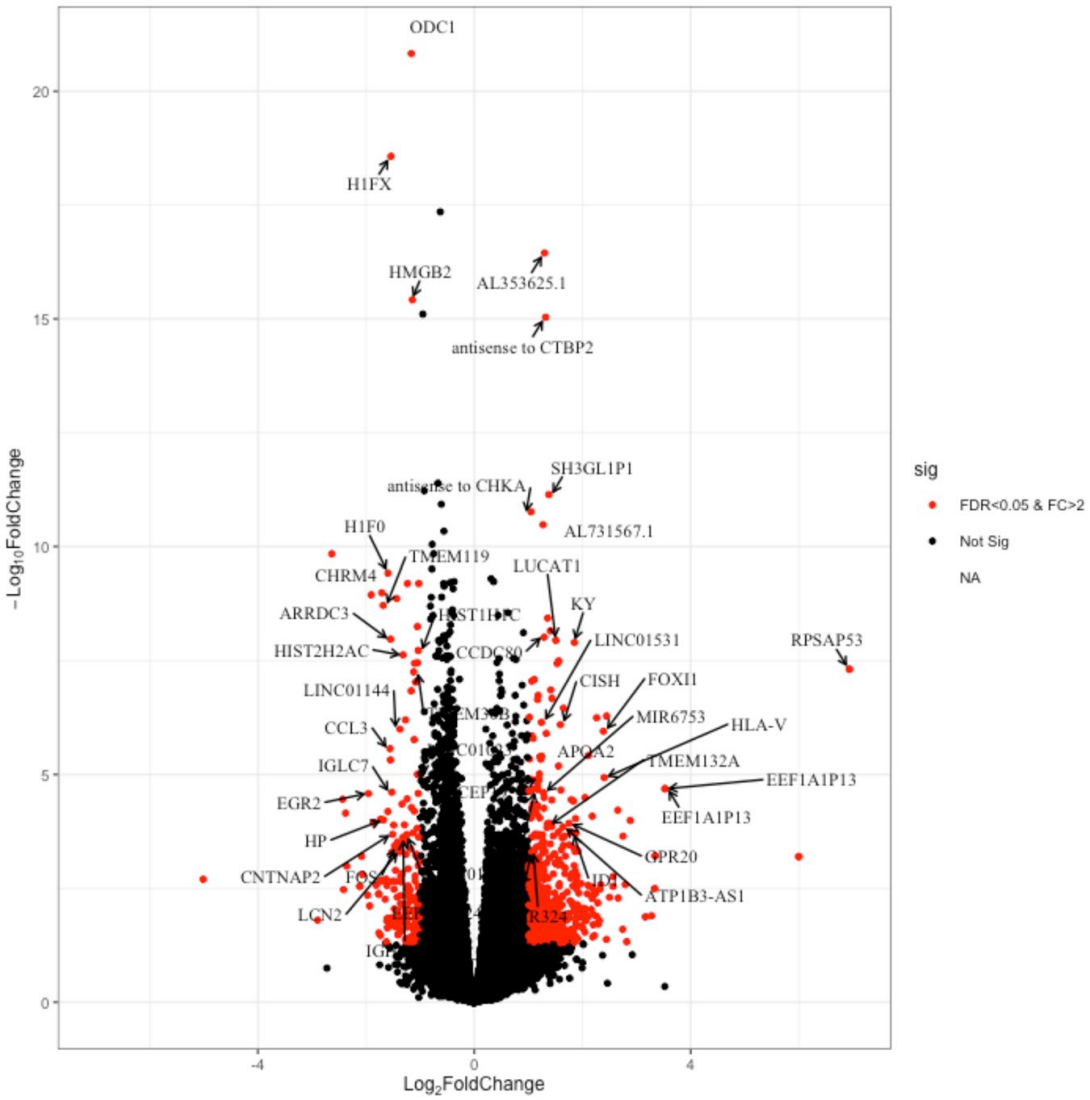
Volcano plot demonstrating the differentially expressed genes in HEU and HUU infants. The x-axis represents the log_2_ fold change while the y-axis represents the −log_10_ of BH adjusted p-value (padj) value. The differentially expressed genes are indicated in red (padj, 0.05 & L_2_FC > 2).

Among the DEGs, *ODC1* was the most divergent gene (BH adjusted p-value = 1.49e-21) followed by *H1FX* (Fig. 2). Interestingly, the top most statistically significant up-regulated genes consisted primarily of non-coding genes (ENSG00000213073), antisense genes (ENSG00000273599 and ENSG00000255031), and a pseudogene (ENSG00000266777) (Fig. 2). Other DEGs comprised genes encoding novel transcripts (57/262) (see Supplementary Table S2). Hierarchical clustering of DEGs identified groups of co-expressed genes. Key among the up-regulated cluster of genes were those coding for G-protein coupled receptors (GPCRs) (*HCAR2, HCAR3, FFAR2*), *TNFSF14* and *CISH*. The down-regulated gene clusters consisted of: five epigenetic factors (*H1FX, HMGB2, HIST2H2AC, HIST1H1C, H1F0*), genes that function in transcriptional regulation (two noncoding RNAs; *RNU6–595P, ATP1B3-AS1*), genes involved in the malaria pathway (*THBS1, CYP1B1, SDC2*) and genes involved in neutrophil-mediated immunity (*LCN2, CAMP, HP, MMP8, BPI, LTF*) (Fig. 2 & Supplementary Table S2).

### General overrepresentation of the G- protein coupled receptor family in up-regulated DEGs

**Figure 3:**
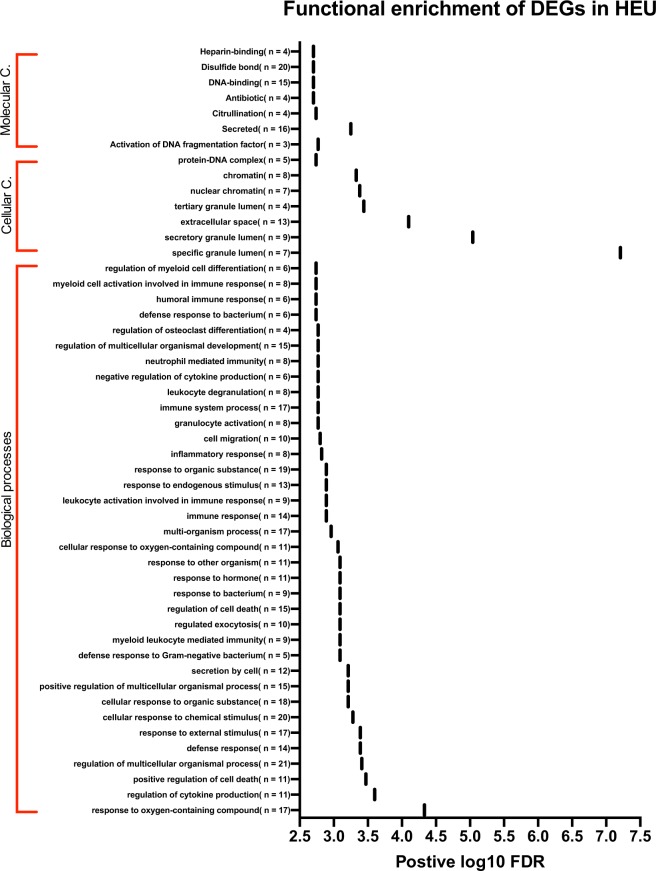
Functional enrichment analysis of the differentially expressed genes (DEGs). The y-axis represents enriched GO terms (FDR < 0.01) associated with DEGs in HEU infants sorted by biological processes, cellular component “Cellular C.” and molecular function “Molecular F.” The x-axis represents the absolute log_10_ FDR values.

To investigate potential molecular and biochemical pathways in gene sets of either up-regulated or down-regulated DEGs, we performed enrichment analysis using STRING^24,25^. In the analysis of the up-regulated gene set, two-thirds of all DEGs surprisingly collapsed into one GO term of plasma membrane, particularly the hydroxycarboxylic acid-binding receptors that belong to the G- protein-coupled receptor family and tumor necrosis factor that is involved in systemic inflammation (Supplementary Table S3). Of interest, the down-regulated gene set was associated with 237 GO terms and KEGG pathways. The enriched terms covered diverse biological pathways such as early growth response, osteogenesis, cellular response to stress and external stimuli, response to the oxygen-containing compounds DNA synthesis and immune system processes (Fig. 3 & Supplementary Table S3). The enriched GO terms related to immune system processes included; neutrophil mediated immunity, defense response to bacterium, defense response to gram-negative bacteria, myeloid and leukocyte activation involved in immune responses, humoral immune responses, negative regulation of cytokine production, leukocyte degranulation and inflammatory responses.

### Altered immune responses towards malaria in HEU infants

**Figure 4:**
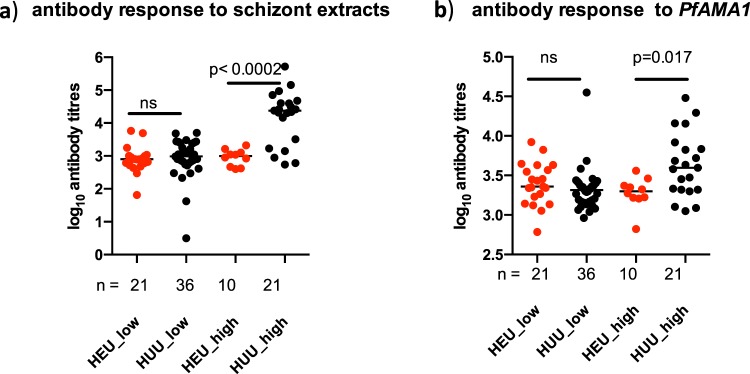
Antimalarial antibody titers in HEU and HUU infants. Antibody responses against (**a**) *Plasmodium* schizont extracts and (**b**) recombinant *plasmodium* apical membrane antigen 1 (PfAMA1). The Mann Whitney non-parametric test was used to compare antibody levels between HEU and HUU infants in high malaria transmission (HEU_high against HUU_high) and between HEU and HUU infants in low malaria transmission areas (HEU_low against HUU_low). Median of log transformed arbitrary antibody tires are shown and p < 0.05 considered significant. HEU; HIV exposed uninfected infants, HUU; HIV unexposed uninfected infants.

Malaria disease was the single KEGG pathway identified and this was linked to *IL6*, syndecan-2 (SDC2) and thrombospondin-1 (THBS1) DEGS that were enriched in the down-regulated gene set. Malaria is endemic in the study region, and thus our data suggests immune responses to the disease may be affected in HEU infants. To test this hypothesis, we analysed antibody responses to *Plasmodium falciparum* apical membrane antigen (*Pf*AMA) 1, a leading malaria vaccine candidate, and responses to *Plasmodium* schizont extract, a crude marker of exposure to malaria parasites during the blood stage infection, in the larger cohort from where the HEU and HUU infants included in the transcriptomic analysis had been sampled. A comparison between the HEU and HUU infants’ antibody titers confirmed the hypothesis. HEU infants residing in a high malaria transmission region, had significantly lower antibodies specific for *Plasmodium* schizont extract (Fig. 4a) and *Pf*AMA1 (Fig. 4b) compared to HUU infants living in the same region. In contrast there were no differences in antibody levels between HEU and HUU infants residing in the low malaria transmission settings, which were similar to those observed in the HEU infants in the high malaria transmission region. Taken together, the antibody responses measured in infants in the larger cohort, support the transcriptional findings in the subset of infants included in the transcriptomic analysis, for the malaria pathway. The low antibody titers in HEU infants corresponds to the low expression of genes coding for host proteins such as *THBS1*, a receptor required for parasite sequestration and avoidance of splenic destruction. In addition to the direct impact of daily cotrimoxazole prophylaxis, it is possible that lower *THBS1*, contributes to adequate splenic clearance of parasites leading to a significant reduction in parasite biomass, further reducing exposure and potentially diminishing antimalarial antibody responses.

### Weighted gene co-expression analysis reveals significant modules in relation to HIV exposure and neutrophils

**Figure 5:**
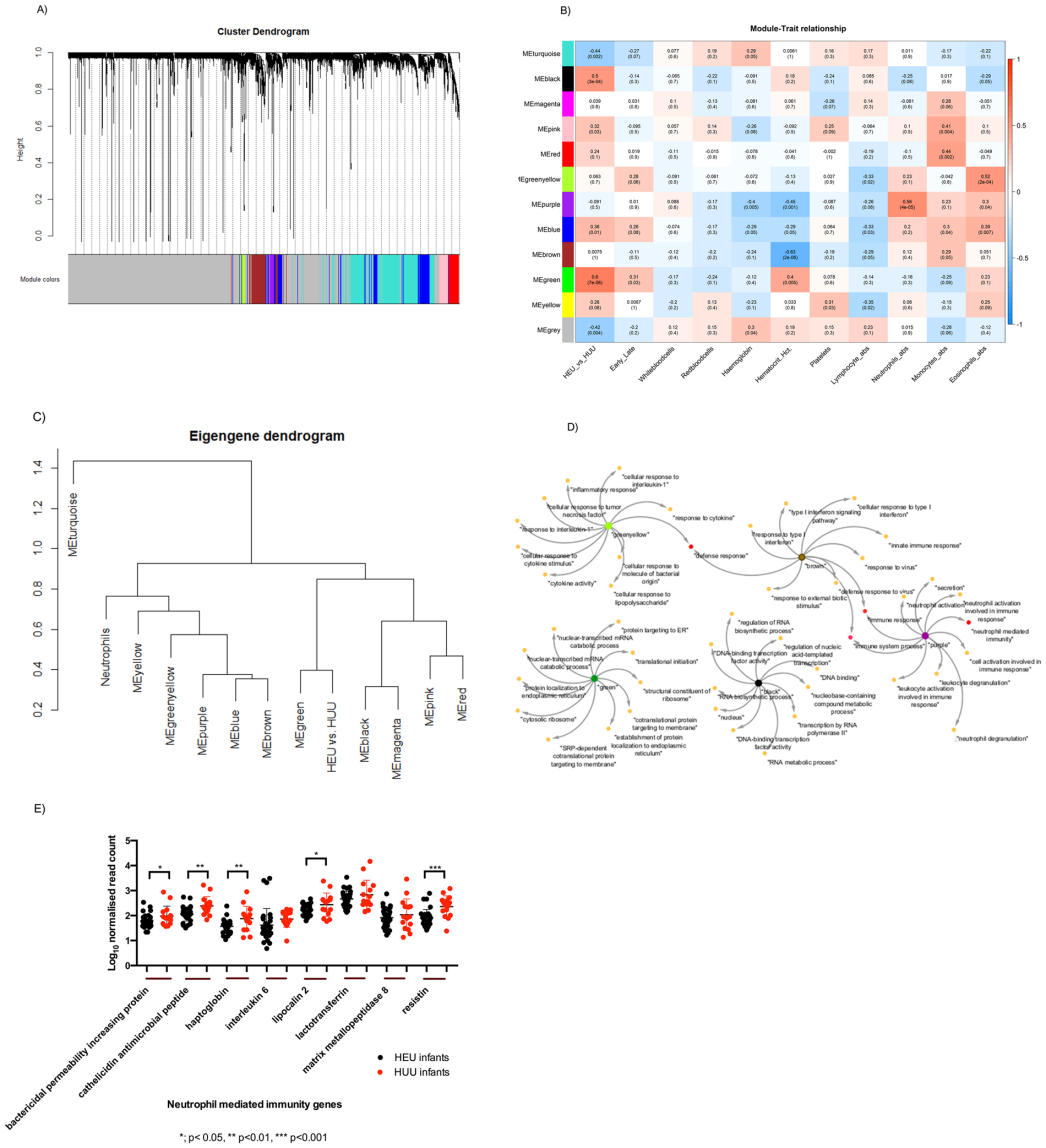
(**A**) Cluster dendogram of co-expression modules. The weighted gene co-expression network analysis identified 12 modules from hierarchical clustering of HEU/HUU co-expression profiles. (**B**) Hierarchical clustering of the module- trait relationship. The y-axis; modules and the x-axis; clinical traits. Pearson’s correlation co-efficient and p-values are shown. Red indicates positive regression while blue indicates negative regression. (**C**) Eigengene network dendogram representing the relationship between the modules and the two traits of interest; HEU vs HUU and neutrophils. (**D**) Gene ontology network representation of the five significant modules; Green, Black, brown, purple and green/yellow. (**E**) Expression analysis of genes involved in neutrophil mediated immunity. The x axis represents genes with significant involvement in neutrophil mediated immunity while the y-axis represents the normalized counts in HEU and HUU infants. *Asterics indicate genes with statistically significant differences (Mann Whitney non-parametric test). HEU; HIV exposed uninfected infants, HUU; HIV unexposed uninfected infants.

To identify groups of genes with similar expression patterns, we performed unsigned WGCNA. Co-expression analysis of 8000 genes from a total of about 17,000 genes resulted in 12 modules of co-expressed genes (Fig. 5A) whereby the grey module contains unassigned genes. Five modules of clinical interest had significant association between the module eigengenes and clinical traits associated with HIV exposure (Fig. 5B). Modules *green (cor* = *0.6, P-value* = *7e-06)* and black *(cor* = *0.5, P-value* = *3e-04)* were significant within the HIV exposure trait (HEU vs HUU) whereas modules brown *(cor* = *0.63, P-value* = *2e-06)*, purple *(cor* = *0.56, P-value* = *4e-05)* and green/yellow *(cor* = *0.52, P-value* = *2e-04)* were significant within the hematocrit, neutrophils and eosinophils traits, respectively (Fig. 5B).

Eigengene network analysis resulted in similar expression patterns between the neutrophil trait and the brown, purple and green/yellow modules, whereas the HEU vs. HUU trait had similar expression patterns to the green and black modules (Fig. 5C).

### Enrichment analysis of DEGs and WGCNA reveals an overrepresentation of neutrophil mediated immunity

Of the five significant modules, modules brown, purple and green/yellow were enriched in immunological GO terms (Fig. 5D & supplementary Table S4). Of interest was the purple module that also had neutrophil mediated immunity as an enriched term (*Bonferroni corrected P-value* = *7.18e-04*) (Supplementary Table S4). Further analysis of individual DEGs linked to neutrophil-mediated immunity revealed a general trend of down-regulated genes in HEU infants (Fig. 5E). The most significant change was observed in the gene coding for resistin (*RETN*), that plays a crucial role in neutrophil activation and the formation of neutrophil extracellular traps (NETs)^26^.

**Figure 6:**
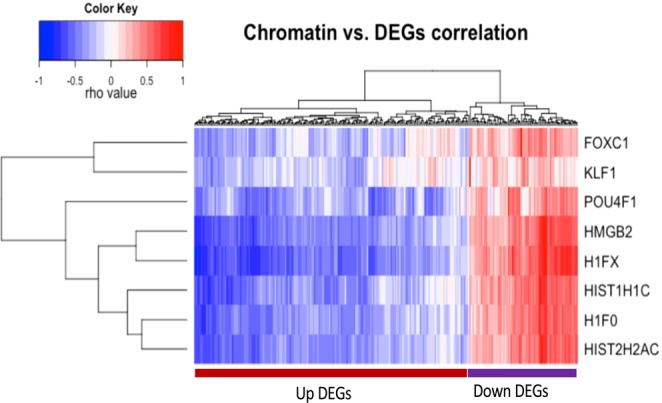
Pearson correlation analysis comparing the transcription profile between chromatin genes and DEGs. Red and blue color-coding indicates relative increase or decrease in gene expression profile, respectively. Differentially expressed genes and Chromatin genes are represented by the x and y axis respectively.

Epigenetic factors have been shown to be regulators of global transcription through chromatin remodelling. In our study *H1FX* and *HMGB2* genes, were top amongst the significantly down-regulated genes in HEU infants. Interestingly, on performing a correlation analysis between the 262 DEGs and individual genes from the chromatin hub, a strong positive correlation with all the downregulated genes and a strong negative association with the upregulated genes was observed (Fig. 6). This observation suggests a potential role for the chromatin-related genes in modulating the dysregulation of the HEU infants’ transcriptome.

## Discussion

Overall, the transcriptome analysis draws attention to a specific immune system process that is downregulated in HEU infants, neutrophil mediated immunity, that could be a potential marker of increased susceptibility to infection and resulting morbidity and mortality.

It is speculated that viral particles may traverse the placental membrane, most likely due to the trans-placental diffusion of HIV soluble proteins as opposed to live and replication-competent HIV. Exposure to HIV soluble proteins may trigger immune responses to HIV. Previous reports showed that more than 20% of HEU infants possess immunological memory to HIV despite not being infected^27^, suggesting that early exposure may cause intrinsic immunological triggers. In this study, we observed the upregulation of non-coding RNA (ncRNA) *ATP1B3-AS1*, in HEU infants. *ATP1B3-AS1* is an antisense transcript of *ATP1B3* gene, which belongs to the Na^+^/K^+^ ATPase family that catalyzes the hydrolysis of ATP. The *ATP1B3* gene has recently been shown to be a cofactor of the bone marrow stromal cell antigen 2 (BST-2) protein. BST-2 is highly expressed on the surface of HIV infected cells and has been demonstrated to stop the release of newly produced virions by blocking virus escape from infected cells. Knockdown of *ATP1B3* enhances the expression of BST-2, in turn modulating the restriction of HIV-1 virion production. The high expression of the antisense transcript, *ATP1B3-AS1*, may therefore be an indicator of exposure to viral particles and the priming of BST-2 dependent anti-HIV responses in HEU infants as previously suggested^28^.

The high expression of genes primarily associated with the plasma membrane identified a number of receptor genes including those on the surface of immune cells. The high transcription levels of *TNFSF14, CISH, FFAR2, HCAR2*, and *HCAR3* in HEU infants, highlights a role for immune processes involving cells such as dendritic cells (DCs), neutrophils and macrophages. *TNFSF14* is expressed on activated T lymphocytes, preferentially CD8+^29^, and the upregulation of *TNFSF14* could be as a result of increased CD8+ activation as previously reported in HEU infants^17,30^*. CISH* is expressed during the development of DCs and regulates DC-mediated cytotoxic T-lymphocyte activation^31,32^. Its expression is associated with the negative regulation of cytokine signaling, controlling inflammation during infection, and susceptibility to infectious diseases such as bacteria and malaria^33^. High expression of *CISH* in HEU infants may similarly predispose them to severe bacterial infection and may be potentially associated with the high inflammation observed in HEU infants^34^. *HCAR2* and *HCAR3*, encode for receptors expressed on various immune cells such as neutrophils and macrophages, which have been shown to have anti-inflammatory effects^35–38^. *FFAR2*, on the other hand, regulates the differentiation and activation of macrophages and leukocytes^39^.

Further analysis of the downregulated genes revealed a high concentration of DEGs related to immune processes associated with neutrophils. Our data has demonstrated the down-regulation of several genes coding for proteins involved in chromatin structure and regulation of the global transcription program. The strong co-regulation of expression between the DEGs and the chromatin genes, implies that they both contribute to the observed transcriptome changes in HEU infants and identified biological pathways. However, we acknowledge that there are likely to be additional players to the chromatin genes that may contribute to the observed changes. Indeed, several non-coding RNA species including *LUCAT1* and *LINC01531* were among the DEGs that could potentially be involved in transcriptional regulation.

HEU infants have been reported to have normal neutrophil counts with an impaired neutrophil function^40^. Neutrophils function in both the innate and adaptive immune system^41,42^ attacking microorganisms by phagocytosis, degranulation or via the generation of NETs^43–45^. Genes primarily associated with neutrophil mediated immunity (*LCN2, CAMP, HP, RETN, MMP8, BPI, LTF*) were repressed in the HEU infants and could be a contributing factor to the increased susceptibility of HEU infants to bacterial infections^46–48^. *RETN*, found in specific neutrophil granules and cell membranes^49^ plays a role in the formation of NETs and the activation of neutrophils^26^, whereas *BPI* is bactericidal and stimulates phagocytosis by neutrophils through the complement system^50^. *LCN2* encodes for lipocalin 2 (neutrophil gelatinase-associated lipocalin - NGAL), a protein mostly released by neutrophils. This protein is known for its bacteriostatic role^46^ and enhancement of phagocytosis by macrophages^51^. The downregulation of *HP* which encodes for an acute phase bacteriostatic protein that is released during inflammation and injury by activated neutrophils^52^, may also interfere with neutrophil function. *CAMP* encodes for cathelicidin antimicrobial peptides which not only have direct antimicrobial^44,53^ properties, but they also affect neutrophil activity through neutrophil recruitment^54,55^ and inhibition of neutrophil apoptosis^56,57^.

It is worth noting that the HEU infants in this study were on daily cotrimoxazole prophylaxis in line with the recommended policy to protect them from *Pneumocystis jirovecii* pneumonia and they had regular clinical visits, which ruled out on-going febrile infections during blood sample collection. Whether daily cotrimoxazole is beneficial and reduces invasive bacterial infections^58^ is unclear as evidence for this is weak and current guidelines have been debated^59,60^. Some studies have reported little to no effect of cotrimoxazole on mortality, LRTIs and diarrheal infections^60–62^, while others have observed a significant reduction in hospitalizations^63^. Cotrimoxazole not only acts as an antimicrobial agent, it may also directly interfere with the host’s immune system^64^. Therefore, we cannot rule out the immunomodulatory role of cotrimoxazole on the gene expression profiles observed in HEU infants and a repeat of the analysis post cotrimoxazole prophylaxis, that is, after two years of life, may be important for future work. Daily cotrimoxazole may also explain the lower expression profiles for genes associated with malaria pathogenesis on the KEGG pathway. Although not the primary aim, daily cotrimoxazole does protect against malarial disease as previously shown^65–67^. In the wider cohort, HEU infants residing in a high malaria transmission area had lower antibody levels to *Plasmodium* schizont extract and *Pf*AMA1 compared to the community controls living in the same region, implying that they had experienced fewer malaria episodes, in agreement with the gene expression profile.

To our knowledge, this is the first study that has used a transcriptomic approach to describe the gene expression profile of PBMCs from HEU infants. Our data confirm previous reports indicating reduced immune responses in HEU infants^4,7,13,14,20^, as well as revealing new insights into the potential pathways involved in the global dysregulation of immune development in this group of infants. We observed changes in genes involved in innate immune responses, specifically neutrophil function. It is possible that HEU infants have an ineffective neutrophil response that would predispose them to severe bacterial infections and subsequently increased morbidity and mortality, and further studies are warranted to address this.

## Materials and Methods

All methods were performed in accordance with the Kenya Medical Research Institute Scientific and Ethics Review Unit guidelines and regulations for handling and analyzing human-derived biological samples. The Kenya Medical Research Institute Scientific and Ethics Review Unit provided ethical approval (SSC2085) and written informed consent was obtained from mothers of the infants recruited into the study.

### Study site and participants

This study used samples previously collected from a longitudinal cohort of HEU infants recruited between 2011 and 2012 at the comprehensive care and research clinic (CCRC), Kilifi County hospital, previously described^22,23^. HIV care was provided as per the World Health Organization recommendation at the time of the study. Briefly, HEU infants born to mothers, not on HAART were prescribed nevirapine prophylaxis at birth. Treatment was continued until one week after the complete cessation of breastfeeding, while for those with mothers on HAART, nevirapine prophylaxis was stopped at six weeks of life. Infant’s HIV-1 status was determined by polymerase chain reaction (PCR) at six weeks of age or at first contact, and repeated by a rapid antibody test at nine and 18 months of age. Infants suffering from an acute infection or malnutrition at the time of recruitment were excluded from the study. The infants were followed up longitudinally every three months until they were 24 months of age, with an upper age limit of up to 30 months being used to cover late follow-up visits. All HEU infants were given prophylactic cotrimoxazole during the first 18 months of life. Age-matched community control HUU infants were recruited from three localities within the catchment area of Kilifi County with varying malaria endemicity and sampled at a single time point^20,22^. From the parent study, we used samples from 19 HEU infants that contributed to the 12 and 24-month time points. However, one and five infant samples were not available at the 12 and 24-month time points, respectively. Also, we obtained control samples from 15 HUU infants specifically from the low malaria transmission region, to avoid any malaria interference on gene expression profiles. Due to ethical reasons, we could not get blood samples from healthy HUU infants more than once. Samples from selected age-matched infants at 12 months or 24 months were therefore analyzed. In total 34 individuals (19 HEU infants matched to 15 HUU infants) were used for RNA sequencing and the gene expression profiling reported here. During the parent study, 5 ml whole blood was drawn and processed within 4 hours. From this, 300 µl of whole blood was used for RNA isolation by first depleting the red blood cells through lysis and storing the PBMCs at −80 °C until ready for RNA isolation.

### Bioinformatics analysis

#### mRNA library preparation, sequencing and quality control

The frozen PBMCs were thawed in bulk and RNA extracted as per the Qiagen RNeasy kit protocol. The quality of the extracted RNA was confirmed using Agilent Bioanalyzer and messenger RNA (mRNA) enrichment done. mRNA sequencing libraries for 200 bp short-inserts were prepared according to the manufacturer’s recommendations. RNA sequencing was done using the Hi-Seq Illumina platform using nine lanes of single-ended reads at a read length of 50 base-pairs (50 bp). RNA extraction, Library preparation, and sequencing were performed at the Beijing Genomics Institute (BGI), China.

#### Genome assembly and abundance estimation

High quality sequenced data was obtained through quality control which involved removing the adapter sequences, low quality reads and unknown sequences. Using Tophat2 v2.1.051^68^ and bowtie2 v2.2.552^69^ software, the high quality reads were aligned against the human reference genome GRCh38vs86 obtained from Ensembl^70^ using the file transfer protocol (FTP). The estimated abundance levels were then quantified at the exon level using HTSeq v0.6.154^71^. The output of abundance estimation was in form of text files for each sample. Text files from all samples were combined into a count matrix for differential expression analysis using Bioconductor packages in R.

#### Differential expression analysis

The quantified abundance estimates, in the form of a count matrix, were normalized and analyzed for differential gene expression using the geometric mean method and negative binomial distribution incorporated in the DESeq2 v1.22.2 package^72^. A threshold of Padj < 0.05, after adjusting for multiple testing using Benjamini-Hochberg, and an absolute log2 fold change of 1 was used to identify differentially expressed genes (DEGs). Hierarchical clustering analysis was then done on the DEGs.

#### Unsigned weighted gene co-expression networks

Unsigned weighted gene network analysis was performed using the Weighted gene co-expression network analysis (WGCNA) R package^73^. As input data, variance stabilizing transformation was applied to the expression dataset using the variance stabilization transformation function in the DESeq2 package. Genes with read counts less than 10 and “NA” as a p-value were filtered out and using a heuristic cut-off, the top 8000 most variant genes were selected. Correlational analysis of the selected genes was done using the Pearson correlation co-efficient to create a similarity matrix. Power adjacency function (*β*) was used for soft thresholding to convert the similarity matrix into an adjacency matrix. Scale free topology criterion was used to obtain the power adjacency function and linear regression model fitting index *R*^2^ used to choose the optimum power for our analysis, *β* = 20 (supplementary Fig. S4). An unsigned topological overlap matrix with a minimum module size of 30 and a merge cut height of 0.25 was used to create co-expression gene modules from the adjacency matrix. The gene modules were assigned colors and visualized using a hierarchical clustering dendogram.

#### Module-trait relationship

The module-trait relationship was determined by relating eigengenes from each module to clinical traits using pearson correlation. Associations were deemed significant if the eigengene module membership had a p-value of ≤0.001 and the eigengene-trait absolute cor was ≥0.25. The significant modules were further analysed for functional enrichment.

#### Functional enrichment analysis

We conducted enrichment analysis on the significant DEGs using the Search Tool for Retrieval of Interacting Genes/Proteins (STRING) v11.0 software^24^. DEGs were divided into two gene sets, up-regulated and down-regulated, as input and an evidence score of 0.7 selected to increase stringency in the analysis. Enrichment analysis was performed on the significant modules using the *GOenrichmentanalysis* function of WGCNA R package and visualized using Cytoscape^74^.

#### Analysis of antimalarial antibody responses

During the parent study, antibody levels against crude *Plasmodium falciparum* schizont extracts and PfAMA1 had been measured using previously reported protocols^75,76^. Plasma samples from HEU (n = 31) and HUU (n = 57) infants were analysed at 18 months of age (to rule out any maternal antibody influences). Samples were classified based on malaria exposure, that is, HEU and HUU infants residing in high malaria transmission region (n = 10 and n = 21, respectively) or HEU and HUU infants residing in low malaria incidence region in Kilifi (n = 21 and n = 36, respectively). Of the infants whose antibody levels had been determined, HEU (n = 6) and HUU (n = 5) were infants included in the current transcriptome analysis. Only HUU infants from the low malaria transmission region were included in the transcriptome analysis to avoid potential impact of malaria exposure on gene expression profiles. To determine antibody titers, plates pre-coated with 20 ng/µl of recombinant- *Pf*AMA1 or crude *Plasmodium falciparum* schizont extract were probed with sera from HEU infants or controls (HUU) and IgG antibody levels against *Pf*AMA1 or schizont extract measured in optical density (OD) values. Hyperimmune serum serially diluted and included on each plate was assigned antibody arbitrary units and used to convert OD values of the samples into relative arbitrary units. The arbitrary units were then log transformed to normalize the antibody titres and the non-parametric Mann-Whitney test used to determine differences in median antibody titres between HEU and HUU infants in low, and similarly between HEU and HUU infants in high malaria exposure regions. These data were used to test the observed KEGG pathway enriched in the down-regulated gene set in HEU infants.

## Supplementary information

Supplementary data

Supplementary table S3

Supplementary table S4

## Data Availability

The datasets generated during the current study are available from the corresponding author on reasonable request and will be made available in a public repository.
