# Supplementary material for: Peripheral blood mononuclear cell transcriptomes reveal an over-representation of down-regulated genes associated with immunity in HIV-exposed uninfected infants
Source: Sci Rep. 2019 Dec 2;9:18124. doi: 10.1038/s41598-019-54083-4 (PMC6889308; doi:10.1038/s41598-019-54083-4)
Supplement: Supplementary file 1 — Supplementary data [file 41598_2019_54083_MOESM1_ESM.docx]

**Peripheral blood mononuclear cell transcriptomes reveal an over-representation of down-regulated genes associated with immunity in HIV-exposed uninfected infants**.

*Zaneta D. Musimbi^1^, *Martin K. Rono^2,3^, James R. Otieno^2^, Nelson Kibinge^2^, Lynette Isabella Ochola-Oyier^2,3^, Etienne Pierre de Villiers^2,4^ & Eunice W. Nduati^2,3^


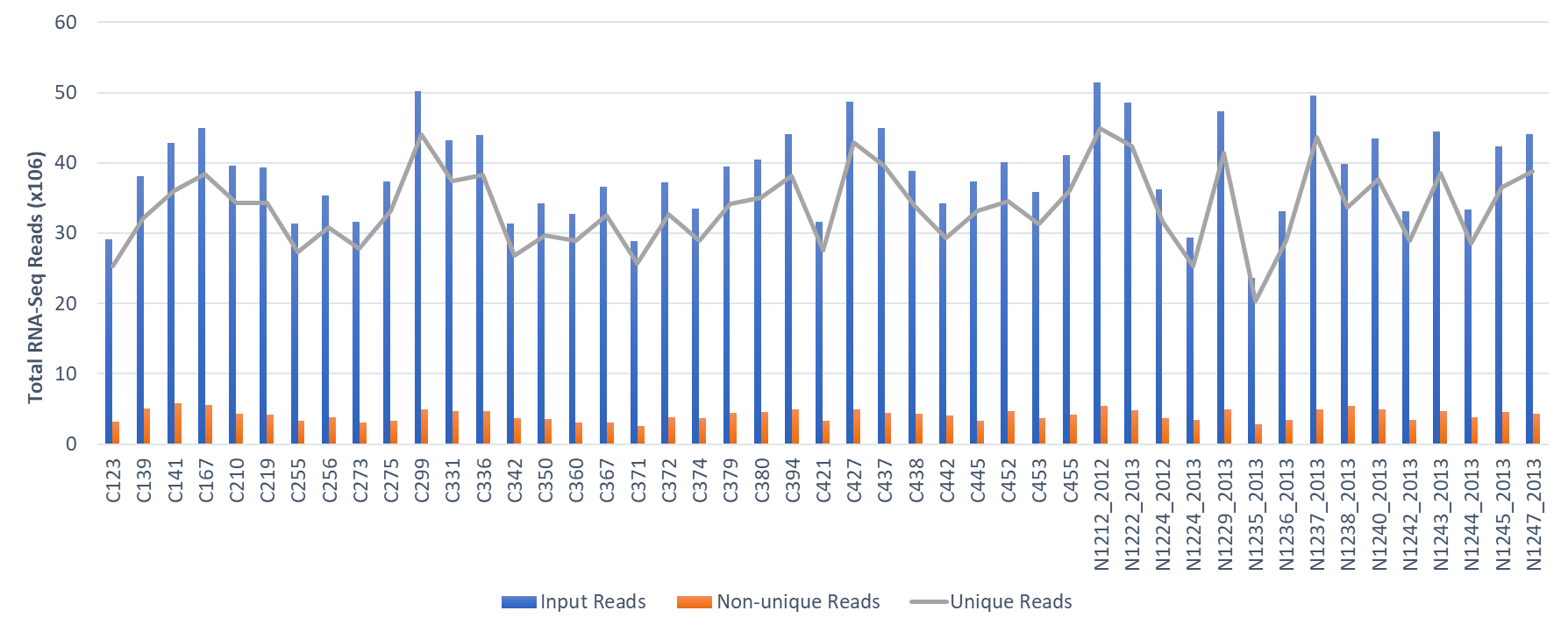


**Supplementary Fig. S1** RNAseq mapping statistics. Bar graph analysis of sequenced reads from HEU and HUU PBMCs were quality checked and mapped against reference human genome predicted transcripts. Blue bars indicate total mapped reads, grey line: reads uniquely mapping to a single transcript, and red bars: reads mapping to multiple transcripts. HEU; HIV-exposed uninfected, HUU; HIV unexposed uninfected, PBMCs: Peripheral blood mononuclear cells.

**
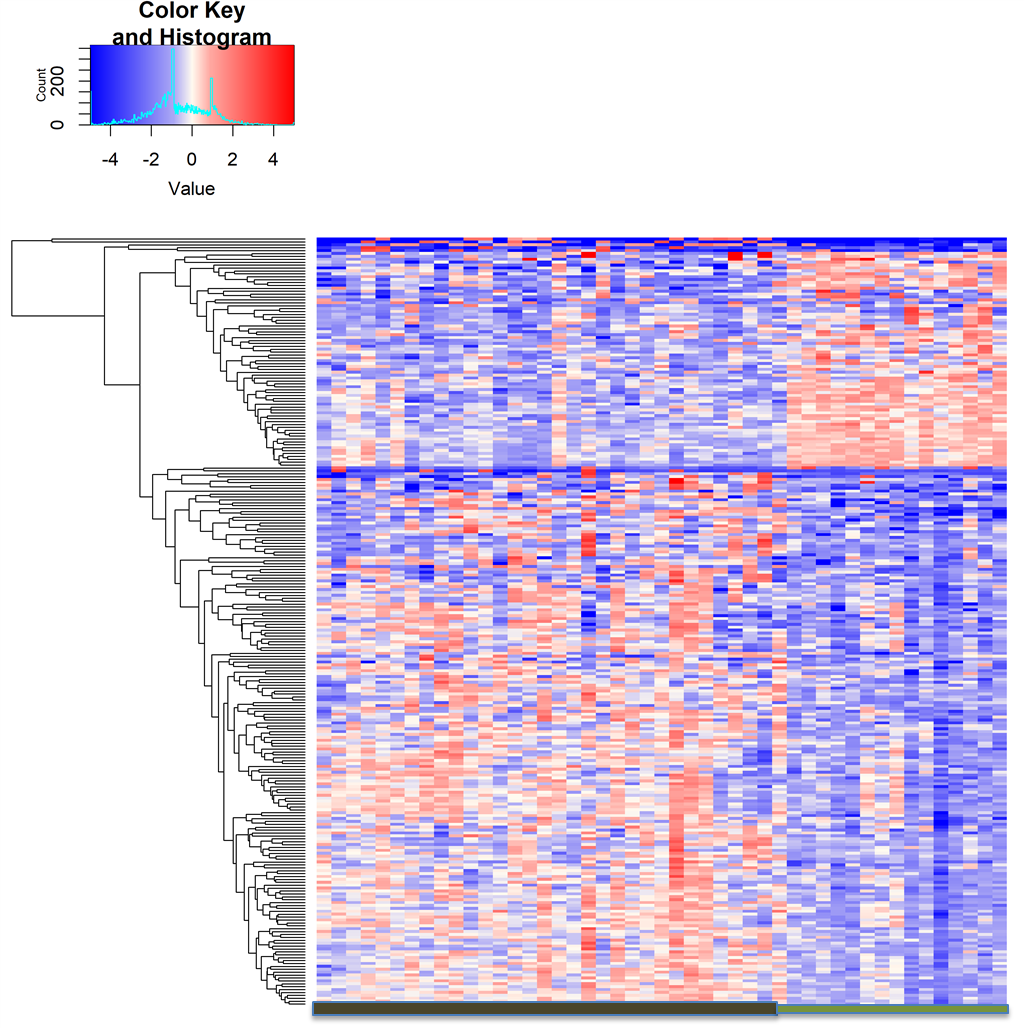
**

**Supplementary Fig. S2** Hierarchical clustering of the 262 differentially expressed genes. Red and blue color-coding indicates relative increase or decrease in gene expression profile, respectively. Samples and DEGs are represented by the x and y axis respectively. HEU and HUU samples are represented by the colours dark brown and green respectively. DEGs: Differentially expressed genes, HEU; HIV-exposed uninfected, HUU; HIV unexposed uninfected.


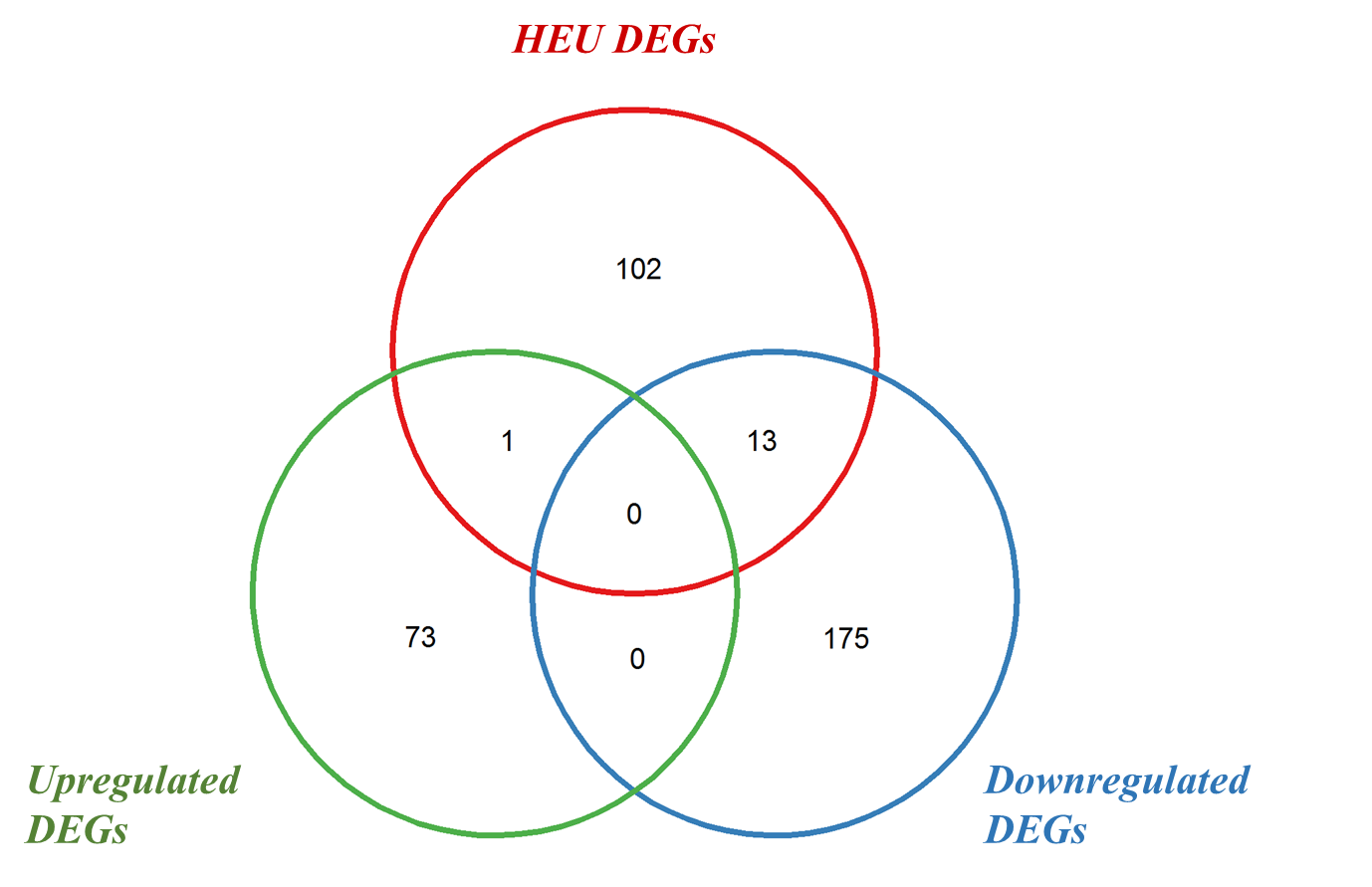


**Supplementary Fig. S3** Venn plot showing an overlap between DEGs in infants of age < 12 months and up- and down-regulated genes in all the infant ages (<24 months). The red circle indicates the DEGs in infants of age <12 months, the blue indicates the downregulated genes and the green circle indicates the upregulated genes in all the infants. DEGs; Differentially expressed genes.


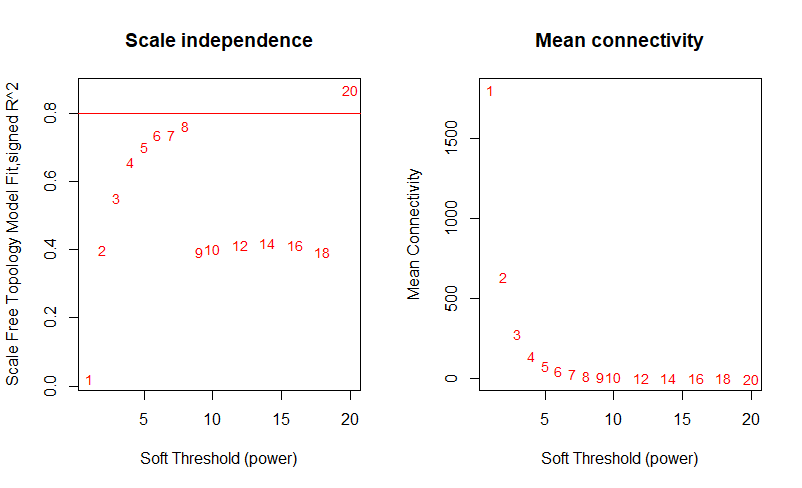


**Supplementary Fig. S4** Soft-thresholding power for scale free topology model. a) Scale independence: the y-axis represents the square of the model fitting index R whose peak is 0.8.and x axis represents the soft threshold. Any power ≥ 0.8 satisfies the scale of free topology. b) Mean connectivity: the y axis represents the mean connectivity under different weighting coefficients

**Supplementary Table S1: Haematological parameters for HIV unexposed uninfected community controls (HUU) and HIV exposed uninfected (HEU) infants**

|  | **HEU infants**  **n = 32** | **HUU infants**  **n = 15** | **p-value** |
| --- | --- | --- | --- |
| **WBC count (10^3^/ul)** | 11.3 [9-14.2] | 9.8 [9-11] | 0.2911 |
| **RBC count (10^6^/ul)** | 4.7 [4.3-5] | 4.6 [4.3-4.9] | 0.4207 |
| **Hb conc (mg/dL)** | 10 [9-11] | 9.7 [8.9-10.2] | 0.3344 |
| **Platelets (10^3^/ul)** | 454 [376-605] | 418 [384-514] | 0.4896 |
| **Lymphocytes absolute (10^3^/ul)** | 7.2 [5.5-9.5] | 6.2 [5.7-6.9] | 0.1913 |
| **Neutrophils absolute (10^3^/ul)** | 2.8 [2.2-3.1] | 2.4 [1.8-3.3] | 0.4506 |
| **Monocytes absolute (10^3^/ul)** | 0.64 [0.50-1.07] | 0.70 [0.58-1.05] | 0.5006 |
| **Eosinophils absolute (10^3^/ul)** | 0.285 [0.17-0.49] | 0.38 [0.23-0.60] | 0.3784 |

Haematological parameters were measured at the time of sample collection in HEU and HUU infants. WBC; white blood cells, RBC; red blood cells, Hb conc; haemoglobin concentration. Values are presented as median [25^th^ and 75^th^ percentile], p<0.05 Mann-Whitney U tests with p<0.05 considered significant.

**Supplementary Table S2: 262 Differentially expressed genes in HIV exposed uninfected (HEU) infants**

| **Gene** | baseMean | L2fc | padj | Gene.name | Gene.description |
| --- | --- | --- | --- | --- | --- |
| **ENSG00000115758** | 3697.0 | -1.2 | 1.49E-21 | ODC1 | ornithine decarboxylase 1 |
| **ENSG00000184897** | 4148.8 | -1.5 | 2.69E-19 | H1FX | H1 histone family member X |
| **ENSG00000213073** | 221.9 | 1.3 | 3.58E-17 | AL353625.1 |  |
| **ENSG00000164104** | 4751.5 | -1.1 | 3.82E-16 | HMGB2 | high mobility group box 2 |
| **ENSG00000273599** | 92.4 | 1.3 | 9.21E-16 | AL731571.1 | novel transcript, antisense to CTBP2 |
| **ENSG00000266777** | 67.6 | 1.4 | 7.26E-12 | SH3GL1P1 | SH3 domain containing GRB2 like 1, endophilin A2 pseudogene 1 |
| **ENSG00000255031** | 143.7 | 1.1 | 1.73E-11 | AP002807.1 | novel transcript, antisense to CHKA |
| **ENSG00000231964** | 79.0 | 1.3 | 3.33E-11 | AL731567.1 |  |
| **ENSG00000189060** | 885.2 | -1.6 | 3.79E-10 | H1F0 | H1 histone family member 0 |
| **ENSG00000272931** | 47.5 | -1.2 | 6.38E-10 | AC099568.2 | novel transcript |
| **ENSG00000198551** | 272.2 | -1.0 | 6.38E-10 | ZNF627 | zinc finger protein 627 |
| **ENSG00000180720** | 31.3 | -1.7 | 1.02E-09 | CHRM4 | cholinergic receptor muscarinic 4 |
| **ENSG00000223547** | 532.5 | -1.4 | 1.36E-09 | ZNF844 | zinc finger protein 844 |
| **ENSG00000183160** | 128.9 | -1.7 | 1.93E-09 | TMEM119 | transmembrane protein 119 |
| **ENSG00000173991** | 108.7 | 1.4 | 3.67E-09 | TCAP | titin-cap |
| **ENSG00000257135** | 44.5 | -1.1 | 5.61E-09 | AC007249.2 | novel transcript |
| **ENSG00000218809** | 157.4 | 1.4 | 6.94E-09 | AL391903.1 | triggering receptor expressed on myeloid cells 1 (TREM1) pseudogene |
| **ENSG00000091986** | 105.0 | 1.3 | 9.63E-09 | CCDC80 | coiled-coil domain containing 80 |
| **ENSG00000113369** | 5480.5 | -1.5 | 1.06E-08 | ARRDC3 | arrestin domain containing 3 |
| **ENSG00000248323** | 258.3 | 1.5 | 1.13E-08 | LUCAT1 | lung cancer associated transcript 1 |
| **ENSG00000174611** | 71.3 | 1.9 | 1.26E-08 | KY | kyphoscoliosis peptidase |
| **ENSG00000187837** | 598.5 | -1.0 | 1.88E-08 | HIST1H1C | histone cluster 1 H1 family member c |
| **ENSG00000184260** | 24.0 | -1.3 | 2.35E-08 | HIST2H2AC | histone cluster 2 H2A family member c |
| **ENSG00000125735** | 479.4 | 1.6 | 3.20E-08 | TNFSF14 | TNF superfamily member 14 |
| **ENSG00000174721** | 66.6 | -1.1 | 3.51E-08 | FGFBP3 | fibroblast growth factor binding protein 3 |
| **ENSG00000251867** | 82.6 | -1.1 | 3.61E-08 | AC009812.1 | novel transcript, antisense to ZBTB10 |
| **ENSG00000224950** | 43.0 | 1.5 | 3.66E-08 | AL390066.1 | novel transcript |
| **ENSG00000214263** | 129.8 | 6.9 | 4.86E-08 | RPSAP53 | ribosomal protein SA pseudogene 53 |
| **ENSG00000182107** | 42.2 | -1.1 | 5.57E-08 | TMEM30B | transmembrane protein 30B |
| **ENSG00000234290** | 759.6 | 1.1 | 8.16E-08 | AC116366.1 | novel transcript |
| **ENSG00000126861** | 78.0 | 1.1 | 8.87E-08 | OMG | oligodendrocyte myelin glycoprotein |
| **ENSG00000146072** | 178.0 | -1.1 | 8.87E-08 | TNFRSF21 | TNF receptor superfamily member 21 |
| **ENSG00000135116** | 471.6 | -1.1 | 9.37E-08 | HRK | harakiri, BCL2 interacting protein |
| **ENSG00000279821** | 89.5 | 1.4 | 1.39E-07 | AC145098.2 | TEC |
| **ENSG00000268355** | 36.9 | -1.2 | 1.45E-07 | AC243960.3 | novel transcript |
| **ENSG00000276842** | 180.2 | 1.2 | 1.83E-07 | AC023510.2 | novel transcript, antisense to LRMP |
| **ENSG00000169242** | 58.6 | 1.4 | 2.12E-07 | EFNA1 | ephrin A1 |
| **ENSG00000277595** | 27.9 | 1.2 | 2.25E-07 | AC007546.1 | novel transcript, sense intronic to ANKRD13A |
| **ENSG00000260145** | 21.6 | 1.6 | 3.50E-07 | AC023825.2 | novel transcript, antisense to NLRC5 |
| **ENSG00000256249** | 11.3 | 2.4 | 5.19E-07 | AC026333.3 | novel transcript |
| **ENSG00000235217** | 123.4 | 1.0 | 5.54E-07 | TSPY26P | testis specific protein Y-linked 26, pseudogene |
| **ENSG00000275494** | 14.3 | -1.3 | 6.33E-07 | AC133552.5 | novel transcript |
| **ENSG00000205786** | 21.7 | 1.2 | 7.15E-07 | LINC01531 | long intergenic non-protein coding RNA 1531 |
| **ENSG00000114737** | 851.1 | 1.6 | 8.05E-07 | CISH | cytokine inducible SH2 containing protein |
| **ENSG00000281912** | 16.7 | -1.4 | 1.01E-06 | LINC01144 | long intergenic non-protein coding RNA 1144 |
| **ENSG00000168269** | 13.7 | 2.4 | 1.12E-06 | FOXI1 | forkhead box I1 |
| **ENSG00000238279** | 29.6 | 1.3 | 1.25E-06 | BX470102.1 | novel transcript |
| **ENSG00000279191** | 36.2 | 1.1 | 1.42E-06 | AC068491.4 | TEC |
| **ENSG00000279789** | 89.7 | 1.1 | 1.61E-06 | AC120114.3 | novel transcript, sense intronic to KCTD13 |
| **ENSG00000272583** | 16.4 | -1.1 | 1.71E-06 | AL592494.3 | novel transcript |
| **ENSG00000277632** | 173.8 | -1.6 | 2.70E-06 | CCL3 | C-C motif chemokine ligand 3 |
| **ENSG00000258086** | 12.0 | 2.1 | 3.83E-06 | AC079313.1 |  |
| **ENSG00000250254** | 15.3 | 1.2 | 3.95E-06 | PTTG2 | pituitary tumor-transforming 2 |
| **ENSG00000229808** | 44.8 | 1.2 | 4.08E-06 | AL391825.1 | proteasome (prosome, macropain) activator subunit 3 (PA28 gamma; Ki) pseudogene |
| **ENSG00000251194** | 40.8 | 1.0 | 4.57E-06 | AL133330.1 | novel transcript |
| **ENSG00000218073** | 17.3 | 1.2 | 4.63E-06 | AL021407.3 | PERP, TP53 apoptosis effector (PERP) pseudogene |
| **ENSG00000169715** | 59.1 | -1.5 | 4.79E-06 | MT1E | metallothionein 1E |
| **ENSG00000158874** | 30.2 | 1.6 | 6.51E-06 | APOA2 | apolipoprotein A2 |
| **ENSG00000166289** | 672.3 | 1.2 | 9.74E-06 | PLEKHF1 | pleckstrin homology and FYVE domain containing 1 |
| **ENSG00000273148** | 117.2 | -1.1 | 9.89E-06 | AL035563.1 |  |
| **ENSG00000272523** | 26.8 | -1.0 | 1.06E-05 | LINC01023 | long intergenic non-protein coding RNA 1023 |
| **ENSG00000181126** | 128.1 | 2.4 | 1.17E-05 | HLA-V | major histocompatibility complex, class I, V (pseudogene) |
| **ENSG00000174007** | 286.8 | 1.2 | 1.23E-05 | CEP19 | centrosomal protein 19 |
| **ENSG00000150048** | 44.6 | 1.0 | 1.28E-05 | CLEC1A | C-type lectin domain family 1 member A |
| **ENSG00000065621** | 93.9 | 1.1 | 1.35E-05 | GSTO2 | glutathione S-transferase omega 2 |
| **ENSG00000188152** | 23.9 | 1.2 | 1.35E-05 | NUTM2G | NUT family member 2G |
| **ENSG00000008323** | 51.0 | 1.2 | 1.73E-05 | PLEKHG6 | pleckstrin homology and RhoGEF domain containing G6 |
| **ENSG00000250182** | 1175.8 | 3.5 | 2.05E-05 | EEF1A1P13 | eukaryotic translation elongation factor 1 alpha 1 pseudogene 13 |
| **ENSG00000246451** | 112.9 | 1.1 | 2.09E-05 | AL049840.1 | novel transcript, antisense to KLC1 |
| **ENSG00000228329** | 37.6 | 1.2 | 2.13E-05 | LINC01890 | long intergenic non-protein coding RNA 1890 |
| **ENSG00000278937** | 18.5 | 1.6 | 2.20E-05 |  | lncRNA |
| **ENSG00000260114** | 66.0 | 1.0 | 2.26E-05 | *CTD2574D22.4* | lncRNA |
| **ENSG00000135437** | 40.8 | 1.0 | 2.36E-05 | RDH5 | retinol dehydrogenase 5 |
| **ENSG00000211685** | 123.7 | -1.5 | 2.46E-05 | IGLC7 | immunoglobulin lambda constant 7 |
| **ENSG00000275022** | 19.3 | 1.3 | 2.52E-05 | MIR6753 | microRNA 6753 |
| **ENSG00000139832** | 692.2 | -1.0 | 2.60E-05 | RAB20 | RAB20, member RAS oncogene family |
| **ENSG00000122877** | 604.6 | -2.0 | 2.62E-05 | EGR2 | early growth response 2 |
| **ENSG00000267293** | 16.4 | 1.3 | 3.19E-05 | AC012569.1 | U3 small nucleolar RNA-associated protein 18 homolog, pseudogene |
| **ENSG00000242265** | 299.7 | -1.2 | 3.42E-05 | PEG10 | paternally expressed 10 |
| **ENSG00000255893** | 14.4 | 1.4 | 3.64E-05 | AP000786.1 | novel transcript |
| **ENSG00000198711** | 59.6 | 1.1 | 4.56E-05 | SSBP3-AS1 | SSBP3 antisense RNA 1 |
| **ENSG00000239653** | 224.1 | 1.2 | 4.78E-05 | PSMD6-AS2 | PSMD6 antisense RNA 2 |
| **ENSG00000272037** | 20.5 | 1.2 | 5.20E-05 | AP002907.1 | novel transcript, antisense to UBR5 |
| **ENSG00000163053** | 39.6 | -1.2 | 5.31E-05 | SLC16A14 | solute carrier family 16 member 14 |
| **ENSG00000143858** | 16.6 | 1.3 | 5.52E-05 | SYT2 | synaptotagmin 2 |
| **ENSG00000267519** | 740.7 | 1.1 | 5.60E-05 | AC020916.1 | novel transcript |
| **ENSG00000143257** | 27.2 | 1.1 | 6.08E-05 | NR1I3 | nuclear receptor subfamily 1 group I member 3 |
| **ENSG00000205426** | 14.3 | 2.7 | 6.10E-05 | KRT81 | keratin 81 |
| **ENSG00000162552** | 13.6 | 1.2 | 7.28E-05 | WNT4 | Wnt family member 4 |
| **ENSG00000279187** | 18.2 | 1.2 | 8.96E-05 | AC027601.5 | TEC |
| **ENSG00000164707** | 43.1 | 1.1 | 8.98E-05 | SLC13A4 | solute carrier family 13 member 4 |
| **ENSG00000241157** | 10.3 | 1.9 | 9.29E-05 | AC104763.1 | ribosomal protein L32 (RPL32) pseudogene |
| **ENSG00000257017** | 77.2 | -1.7 | 9.94E-05 | HP | haptoglobin |
| **ENSG00000267607** | 10.1 | 2.9 | 1.02E-04 | AC011511.5 | novel transcript, antisense to ICAM4 and ICAM1 |
| **ENSG00000118523** | 35.0 | -1.9 | 1.13E-04 | CTGF | connective tissue growth factor |
| **ENSG00000233360** | 28.1 | 1.4 | 1.17E-04 | Z83844.2 |  |
| **ENSG00000204882** | 10.1 | 1.8 | 1.19E-04 | GPR20 | G protein-coupled receptor 20 |
| **ENSG00000274602** | 175.8 | 1.4 | 1.19E-04 | PI4KAP1 | phosphatidylinositol 4-kinase alpha pseudogene 1 |
| **ENSG00000182782** | 1044.0 | 1.3 | 1.22E-04 | HCAR2 | hydroxycarboxylic acid receptor 2 |
| **ENSG00000278356** | 16.6 | -1.3 | 1.27E-04 | AC005911.1 | novel transcript |
| **ENSG00000255398** | 741.9 | 1.4 | 1.27E-04 | HCAR3 | hydroxycarboxylic acid receptor 3 |
| **ENSG00000176641** | 53.0 | -1.5 | 1.29E-04 | RNF152 | ring finger protein 152 |
| **ENSG00000006118** | 45.1 | 1.4 | 1.29E-04 | TMEM132A | transmembrane protein 132A |
| **ENSG00000244124** | 17.6 | 1.6 | 1.43E-04 | ATP1B3-AS1 | ATP1B3 antisense RNA 1 |
| **ENSG00000233668** | 14.0 | 1.3 | 1.46E-04 | AL353662.2 | ribosomal protein S8 (RPS8) pseudogene |
| **ENSG00000152192** | 10.3 | -1.1 | 1.52E-04 | POU4F1 | POU class 4 homeobox 1 |
| **ENSG00000125968** | 116.2 | 1.8 | 1.83E-04 | ID1 | inhibitor of DNA binding 1, HLH protein |
| **ENSG00000279412** | 27.5 | 1.1 | 2.03E-04 | AC020763.3 | novel transcript |
| **ENSG00000253980** | 19.0 | 1.2 | 2.05E-04 | AC010609.1 | novel transcript |
| **ENSG00000174469** | 220.7 | -1.5 | 2.06E-04 | CNTNAP2 | contactin associated protein like 2 |
| **ENSG00000223668** | 18.7 | -1.2 | 2.08E-04 | EEF1A1P24 | eukaryotic translation elongation factor 1 alpha 1 pseudogene 24 |
| **ENSG00000267501** | 15.2 | 1.5 | 2.23E-04 | AC104365.2 | novel transcript |
| **ENSG00000276900** | 359.5 | 1.2 | 2.24E-04 | AC023157.3 | novel transcript, antisense to AMN1 |
| **ENSG00000279396** | 134.5 | 1.2 | 2.27E-04 |  |  |
| **ENSG00000197632** | 171.7 | 2.7 | 2.27E-04 | SERPINB2 | serpin family B member 2 |
| **ENSG00000259556** | 18.1 | 1.1 | 2.36E-04 | AC090971.3 | Leo1, Paf1/RNA polymerase II complex component, homolog (S. cerevisiae) (LEO1) pseudogene |
| **ENSG00000232216** | 481.0 | -1.3 | 2.39E-04 | IGHV3-43 | immunoglobulin heavy variable 3-43 |
| **ENSG00000222009** | 319.4 | 1.3 | 2.95E-04 | BTBD19 | BTB domain containing 19 |
| **ENSG00000262874** | 13.2 | 1.4 | 3.35E-04 | C19orf84 | chromosome 19 open reading frame 84 |
| **ENSG00000213937** | 23.1 | 1.2 | 3.52E-04 | CLDN9 | claudin 9 |
| **ENSG00000126262** | 11486.3 | 1.8 | 3.53E-04 | FFAR2 | free fatty acid receptor 2 |
| **ENSG00000165181** | 18.5 | 1.3 | 3.67E-04 | C9orf84 | chromosome 9 open reading frame 84 |
| **ENSG00000170345** | 13353.5 | -1.4 | 3.81E-04 | FOS | Fos proto-oncogene, AP-1 transcription factor subunit |
| **ENSG00000238025** | 12.1 | 1.8 | 4.12E-04 | ZDHHC4P1 | zinc finger DHHC-type containing 4 pseudogene 1 |
| **ENSG00000239503** | 12.9 | 1.6 | 4.53E-04 | MARK2P8 | microtubule affinity regulating kinase 2 pseudogene 8 |
| **ENSG00000199053** | 21.5 | 1.1 | 4.63E-04 | MIR324 | microRNA 324 |
| **ENSG00000148346** | 281.3 | -1.4 | 4.69E-04 | LCN2 | lipocalin 2 |
| **ENSG00000254554** | 11.4 | 1.3 | 4.75E-04 | AC080023.1 | novel transcript |
| **ENSG00000281332** | 25.0 | 1.1 | 4.82E-04 | LINC00997 | long intergenic non-protein coding RNA 997 |
| **ENSG00000261349** | 30.3 | 1.1 | 4.91E-04 | AL031432.2 | novel pseudogene |
| **ENSG00000233214** | 14.3 | 1.9 | 4.94E-04 | AC002511.2 | novel transcript |
| **ENSG00000164047** | 214.9 | -1.4 | 5.04E-04 | CAMP | cathelicidin antimicrobial peptide |
| **ENSG00000236567** | 31.8 | 1.3 | 5.12E-04 | TCF3P1 | transcription factor 3 pseudogene 1 |
| **ENSG00000269028** | 77.9 | -1.5 | 5.16E-04 | MTRNR2L12 | MT-RNR2 like 12 |
| **ENSG00000138131** | 17.9 | 1.0 | 5.56E-04 | LOXL4 | lysyl oxidase like 4 |
| **ENSG00000230225** | 11.0 | 1.5 | 5.57E-04 | MTND5P14 | MT-ND5 pseudogene 14 |
| **ENSG00000136244** | 53.4 | -1.3 | 5.66E-04 | IL6 | interleukin 6 |
| **ENSG00000223886** | 20.3 | -1.1 | 5.67E-04 | AC073073.1 | ring finger protein 181 (RNF181) pseudogene |
| **ENSG00000224858** | 14.5 | 3.3 | 6.11E-04 | RPL29P11 | ribosomal protein L29 pseudogene 11 |
| **ENSG00000211966** | 1704.3 | -1.1 | 6.12E-04 | IGHV5-51 | immunoglobulin heavy variable 5-51 |
| **ENSG00000181778** | 11.8 | 1.7 | 6.19E-04 | TMEM252 | transmembrane protein 252 |
| **ENSG00000249072** | 15.4 | 6.0 | 6.37E-04 | AC114801.3 | novel pseudogene |
| **ENSG00000267534** | 158.5 | 1.2 | 6.55E-04 | S1PR2 | sphingosine-1-phosphate receptor 2 |
| **ENSG00000267990** | 10.1 | 1.1 | 7.23E-04 | AC063977.2 | novel sialic acid binding Ig-like lectin (SIGLEC) pseudogene |
| **ENSG00000245148** | 81.3 | 1.1 | 7.43E-04 | ARAP1-AS2 | ARAP1 antisense RNA 2 |
| **ENSG00000233030** | 14.4 | 1.6 | 7.45E-04 | AC243772.2 | novel transcript, antisense to FCGR1A |
| **ENSG00000182327** | 23.1 | -1.0 | 7.46E-04 | GLTPD2 | glycolipid transfer protein domain containing 2 |
| **ENSG00000261655** | 19.1 | 1.5 | 8.64E-04 | AC100803.2 | novel transcript, overlapping to GPR20 |
| **ENSG00000229473** | 47.8 | 1.1 | 9.10E-04 | RGS17P1 | regulator of G protein signaling 17 pseudogene 1 |
| **ENSG00000213613** | 27.2 | 1.5 | 9.19E-04 | RPL11P3 | ribosomal protein L11 pseudogene 3 |
| **ENSG00000263624** | 12.0 | 1.3 | 9.63E-04 | AC055811.1 | novel transcript |
| **ENSG00000168229** | 290.5 | 1.0 | 9.93E-04 | PTGDR | prostaglandin D2 receptor |
| **ENSG00000124216** | 134.3 | 1.8 | 1.00E-03 | SNAI1 | snail family transcriptional repressor 1 |
| **ENSG00000279794** | 10.9 | 1.2 | 1.09E-03 | AC024580.1 | TEC |
| **ENSG00000184500** | 78.0 | -1.2 | 1.18E-03 | PROS1 | protein S |
| **ENSG00000177707** | 24.9 | 1.0 | 1.18E-03 | NECTIN3 | nectin cell adhesion molecule 3 |
| **ENSG00000272079** | 12.7 | 1.1 | 1.20E-03 | AC004233.3 | novel transcript |
| **ENSG00000198756** | 79.8 | 1.2 | 1.33E-03 | COLGALT2 | collagen beta(1-O)galactosyltransferase 2 |
| **ENSG00000175556** | 103.1 | 1.2 | 1.63E-03 | LONRF3 | LON peptidase N-terminal domain and ring finger 3 |
| **ENSG00000237161** | 12.8 | 1.3 | 1.63E-03 | AC068446.1 | p21 protein (Cdc42/Rac)-activated kinase 2 (PAK2) pseudogene |
| **ENSG00000261573** | 11.2 | 1.2 | 1.67E-03 | AL157402.2 | novel transcript |
| **ENSG00000163083** | 15.4 | -1.1 | 1.69E-03 | INHBB | inhibin subunit beta B |
| **ENSG00000261594** | 10.7 | -1.4 | 1.70E-03 | TPBGL | trophoblast glycoprotein like |
| **ENSG00000175793** | 67.9 | 1.1 | 1.76E-03 | SFN | stratifin |
| **ENSG00000101665** | 1881.7 | 1.3 | 1.76E-03 | SMAD7 | SMAD family member 7 |
| **ENSG00000124134** | 19.7 | 1.3 | 2.00E-03 | KCNS1 | potassium voltage-gated channel modifier subfamily S member 1 |
| **ENSG00000106328** | 11.2 | 1.0 | 2.08E-03 | FSCN3 | fascin actin-bundling protein 3 |
| **ENSG00000012223** | 1060.3 | -1.7 | 2.15E-03 | LTF | lactotransferrin |
| **ENSG00000104918** | 190.2 | -1.5 | 2.20E-03 | RETN | resistin |
| **ENSG00000063015** | 10.5 | 1.2 | 2.20E-03 | SEZ6 | seizure related 6 homolog |
| **ENSG00000216809** | 16.5 | 1.1 | 2.22E-03 | AL589993.1 | DEAD/H (Asp-Glu-Ala-Asp/His) box polypeptide 24 (DDX24) pseudogene |
| **ENSG00000180846** | 37.7 | 1.1 | 2.34E-03 | CSNK1G2-AS1 | CSNK1G2 antisense RNA 1 |
| **ENSG00000170442** | 42.2 | 1.9 | 2.38E-03 | KRT86 | keratin 86 |
| **ENSG00000271821** | 12.4 | 1.1 | 2.38E-03 | AL662844.3 | novel transcript |
| **ENSG00000279447** | 17.2 | 1.1 | 2.46E-03 | AL118508.4 | TEC |
| **ENSG00000259205** | 61.8 | 1.2 | 2.49E-03 | PRKXP1 | PRKX pseudogene 1 |
| **ENSG00000234292** | 11.1 | 1.2 | 2.50E-03 | AC123595.1 |  |
| **ENSG00000274588** | 28.8 | 1.6 | 2.51E-03 | DGKK | diacylglycerol kinase kappa |
| **ENSG00000236278** | 23.5 | 1.0 | 2.59E-03 | PEBP1P3 | phosphatidylethanolamine binding protein 1 pseudogene 3 |
| **ENSG00000254017** | 35.6 | 1.1 | 2.64E-03 | IGHEP2 | immunoglobulin heavy constant epsilon P2 (pseudogene) |
| **ENSG00000223825** | 10.4 | -1.6 | 2.75E-03 | DAZAP2P1 | DAZ associated protein 2 pseudogene 1 |
| **ENSG00000169439** | 83.9 | -1.2 | 2.80E-03 | SDC2 | syndecan 2 |
| **ENSG00000054598** | 10.7 | -1.0 | 2.82E-03 | FOXC1 | forkhead box C1 |
| **ENSG00000249119** | 37.5 | -2.1 | 2.85E-03 | MTND6P4 | MT-ND6 pseudogene 4 |
| **ENSG00000061656** | 65.1 | 1.1 | 2.88E-03 | SPAG4 | sperm associated antigen 4 |
| **ENSG00000229390** | 17.0 | 2.2 | 2.97E-03 | MICD | MHC class I polypeptide-related sequence D (pseudogene) |
| **ENSG00000251441** | 13.7 | 1.1 | 3.00E-03 | RTEL1P1 | regulator of telomere elongation helicase 1 pseudogene 1 |
| **ENSG00000264304** | 21.2 | 1.1 | 3.22E-03 | AC024267.3 | novel transcript, antisense to FLOT2 |
| **ENSG00000273179** | 110.8 | 1.0 | 3.30E-03 | AC092535.4 | novel transcript, antisense to SPON2 |
| **ENSG00000235128** | 10.0 | 1.1 | 3.40E-03 | AC013474.1 | novel pseudogene |
| **ENSG00000248905** | 345.8 | -1.2 | 3.42E-03 | FMN1 | formin 1 |
| **ENSG00000270911** | 12.6 | 1.1 | 3.44E-03 | AC114878.2 | CTAGE family, member 5 (CTAGE5) pseudogene |
| **ENSG00000177469** | 22.0 | 1.2 | 3.56E-03 | CAVIN1 | caveolae associated protein 1 |
| **ENSG00000237927** | 19.2 | 1.2 | 3.71E-03 | AL078604.2 |  |
| **ENSG00000267560** | 15.1 | 1.0 | 3.88E-03 | AC027514.2 | novel transcript, antisense to KIAA1468 |
| **ENSG00000279811** | 24.9 | 1.0 | 3.93E-03 | AC093330.2 | TEC |
| **ENSG00000231123** | 11.5 | 1.4 | 3.96E-03 | SPATA20P1 | spermatogenesis associated 20 pseudogene 1 |
| **ENSG00000259354** | 23.8 | 1.0 | 4.43E-03 | AC025580.2 | novel transcript, antisense to SLC30A4 |
| **ENSG00000259703** | 10.8 | 1.1 | 4.54E-03 | LINC00593 | long intergenic non-protein coding RNA 593 |
| **ENSG00000254030** | 29.5 | -1.1 | 4.65E-03 | IGLC5 | immunoglobulin lambda constant 5 (pseudogene) |
| **ENSG00000204421** | 12.0 | 1.2 | 4.72E-03 | LY6G6C | lymphocyte antigen 6 family member G6C |
| **ENSG00000269720** | 12.8 | 1.1 | 4.97E-03 | CCDC194 | coiled-coil domain containing 194 |
| **ENSG00000231940** | 11.1 | 1.1 | 5.06E-03 | RPS7P3 | ribosomal protein S7 pseudogene 3 |
| **ENSG00000251230** | 67.4 | 1.5 | 5.54E-03 | MIR3945HG | MIR3945 host gene |
| **ENSG00000237593** | 10.7 | 1.1 | 5.58E-03 | AL445220.1 | ribosomal protein L17 (RPL17) pseudogene |
| **ENSG00000162069** | 29.5 | 1.3 | 5.60E-03 | BICDL2 | BICD family like cargo adaptor 2 |
| **ENSG00000211665** | 53.1 | -1.1 | 6.11E-03 | IGLV3-16 | immunoglobulin lambda variable 3-16 |
| **ENSG00000214872** | 123.2 | 1.1 | 6.37E-03 | SMTNL1 | smoothelin like 1 |
| **ENSG00000275110** | 53.8 | 1.1 | 6.54E-03 | MIR6087 | microRNA 6087 |
| **ENSG00000112139** | 236.9 | 2.3 | 6.58E-03 | MDGA1 | MAM domain containing glycosylphosphatidylinositol anchor 1 |
| **ENSG00000233029** | 29.9 | 1.2 | 6.70E-03 | AC244453.2 | novel transcript |
| **ENSG00000248923** | 67.7 | -1.4 | 6.81E-03 | MTND5P11 | MT-ND5 pseudogene 11 |
| **ENSG00000261026** | 474.6 | 1.0 | 7.21E-03 | AC105046.1 | novel transcript, overlapping to EGR3 |
| **ENSG00000205927** | 23.6 | 1.6 | 7.26E-03 | OLIG2 | oligodendrocyte transcription factor 2 |
| **ENSG00000227502** | 13.5 | 1.5 | 7.28E-03 | LINC01268 | long intergenic non-protein coding RNA 1268 |
| **ENSG00000228463** | 43.8 | -1.0 | 7.29E-03 | AP006222.1 | ribosomal protein L23a (RPL23A) pseudogene |
| **ENSG00000168389** | 249.0 | 1.1 | 7.32E-03 | MFSD2A | major facilitator superfamily domain containing 2A |
| **ENSG00000229512** | 10.4 | 1.1 | 7.38E-03 | AC068580.1 | novel transcript |
| **ENSG00000176170** | 365.6 | 1.0 | 7.38E-03 | SPHK1 | sphingosine kinase 1 |
| **ENSG00000123700** | 776.3 | 1.6 | 7.87E-03 | KCNJ2 | potassium voltage-gated channel subfamily J member 2 |
| **ENSG00000279530** | 54.4 | 1.2 | 8.00E-03 | AC092881.1 | TEC |
| **ENSG00000163661** | 595.3 | 1.5 | 8.01E-03 | PTX3 | pentraxin 3 |
| **ENSG00000260671** | 18.5 | 1.3 | 8.90E-03 | AC010536.2 | novel transcript, sense intronic to KLHDC4 |
| **ENSG00000078401** | 120.1 | 1.3 | 9.20E-03 | EDN1 | endothelin 1 |
| **ENSG00000145975** | 11.3 | 1.0 | 9.29E-03 | FAM217A | family with sequence similarity 217 member A |
| **ENSG00000236772** | 11.3 | 1.0 | 9.67E-03 | AL034550.1 | novel transcript, antisense to NOL4L |
| **ENSG00000250510** | 230.1 | -1.0 | 9.79E-03 | GPR162 | G protein-coupled receptor 162 |
| **ENSG00000101425** | 102.7 | -1.1 | 1.07E-02 | BPI | bactericidal permeability increasing protein |
| **ENSG00000279554** | 19.5 | 1.2 | 1.21E-02 | AC130448.2 | novel transcript |
| **ENSG00000242473** | 15.5 | 1.1 | 1.33E-02 | KIR2DP1 | killer cell immunoglobulin like receptor, two Ig domains pseudogene 1 |
| **ENSG00000105784** | 10.1 | 1.1 | 1.34E-02 | RUNDC3B | RUN domain containing 3B |
| **ENSG00000120738** | 5040.8 | -1.3 | 1.36E-02 | EGR1 | early growth response 1 |
| **ENSG00000138061** | 607.2 | -1.0 | 1.36E-02 | CYP1B1 | cytochrome P450 family 1 subfamily B member 1 |
| **ENSG00000118113** | 190.8 | -1.5 | 1.38E-02 | MMP8 | matrix metallopeptidase 8 |
| **ENSG00000214975** | 13.8 | -1.4 | 1.52E-02 | PPIAP29 | peptidylprolyl isomerase A pseudogene 29 |
| **ENSG00000225690** | 12.7 | 1.1 | 1.54E-02 | TREML5P | triggering receptor expressed on myeloid cells like 5, pseudogene |
| **ENSG00000224083** | 15.3 | 1.1 | 1.55E-02 | MTCO1P11 | MT-CO1 pseudogene 11 |
| **ENSG00000143333** | 142.4 | 1.4 | 1.55E-02 | RGS16 | regulator of G protein signaling 16 |
| **ENSG00000173262** | 54.6 | 1.5 | 1.66E-02 | SLC2A14 | solute carrier family 2 member 14 |
| **ENSG00000200814** | 11.3 | 1.0 | 1.74E-02 | RNU6-595P | RNA, U6 small nuclear 595, pseudogene |
| **ENSG00000211642** | 429.3 | 1.3 | 1.84E-02 | IGLV10-54 | immunoglobulin lambda variable 10-54 |
| **ENSG00000167680** | 140.5 | 1.4 | 1.93E-02 | SEMA6B | semaphorin 6B |
| **ENSG00000253239** | 175.0 | -1.6 | 1.96E-02 | IGLVI-70 | immunoglobulin lambda variable (I)-70 (pseudogene) |
| **ENSG00000236279** | 39.1 | -1.1 | 2.03E-02 | CLEC2L | C-type lectin domain family 2 member L |
| **ENSG00000255221** | 36.7 | 1.1 | 2.13E-02 | CARD17 | caspase recruitment domain family member 17 |
| **ENSG00000211935** | 346.0 | -1.4 | 2.39E-02 | IGHV1-3 | immunoglobulin heavy variable 1-3 |
| **ENSG00000105610** | 16.3 | -1.1 | 2.58E-02 | KLF1 | Kruppel like factor 1 |
| **ENSG00000006327** | 336.2 | 1.2 | 2.67E-02 | TNFRSF12A | TNF receptor superfamily member 12A |
| **ENSG00000133055** | 28.1 | 1.3 | 3.17E-02 | MYBPH | myosin binding protein H |
| **ENSG00000149256** | 14.6 | 1.0 | 3.26E-02 | TENM4 | teneurin transmembrane protein 4 |
| **ENSG00000221957** | 131.7 | 1.0 | 3.40E-02 | KIR2DS4 | killer cell immunoglobulin like receptor, two Ig domains and short cytoplasmic tail 4 |
| **ENSG00000232680** | 20.6 | 1.0 | 3.52E-02 | AC002511.1 | novel transcript |
| **ENSG00000144130** | 27.5 | 1.0 | 3.63E-02 | NT5DC4 | 5'-nucleotidase domain containing 4 |
| **ENSG00000204001** | 59.5 | 2.2 | 3.70E-02 | LCN8 | lipocalin 8 |
| **ENSG00000256050** | 17.5 | -1.2 | 3.75E-02 | AL583722.1 | novel transcript |
| **ENSG00000107130** | 15.4 | 1.1 | 3.89E-02 | NCS1 | neuronal calcium sensor 1 |
| **ENSG00000166450** | 11.2 | 1.3 | 4.07E-02 | PRTG | protogenin |
| **ENSG00000137331** | 1728.5 | 1.2 | 4.31E-02 | IER3 | immediate early response 3 |
| **ENSG00000211625** | 135.2 | 1.3 | 4.41E-02 | IGKV3D-20 | immunoglobulin kappa variable 3D-20 |
| **ENSG00000148344** | 41.9 | 1.1 | 4.74E-02 | PTGES | prostaglandin E synthase |
| **ENSG00000215030** | 1393.1 | -1.2 | 4.74E-02 | RPL13P12 | ribosomal protein L13 pseudogene 12 |
| **ENSG00000137801** | 3811.7 | -1.1 | 4.86E-02 | THBS1 | thrombospondin 1 |
